# Crystallization of metastable monoclinic carnallite, KCl·MgCl_2_·6H_2_O: missing structural link in the carnallite family

**DOI:** 10.1107/S2053229620005197

**Published:** 2020-04-29

**Authors:** Melanie Pannach, Iris Paschke, Horst Schmidt, Daniela Freyer, Wolfgang Voigt

**Affiliations:** a Institut für Anorganische Chemie, TU Bergakademie Freiberg, Leipziger Strasse 29, Freiberg 09599, Germany

**Keywords:** crystallization, metastable, monoclinic, potassium carnallite, crystal structure, potash, fertilizer

## Abstract

During evaporation of natural and synthetic K–Mg–Cl brines, the formation of almost square plate-like crystals of potassium carnallite (potassium chloride magnesium dichloride hexa­hydrate) was observed. A single-crystal structure analysis revealed a monoclinic cell [*a* = 9.251 (2), *b* = 9.516 (2), *c* = 13.217 (4) Å, β = 90.06 (2)° and space group *C*2/*c*]. The structure is isomorphous with other carnallite-type com­pounds, such as NH_4_Cl·MgCl_2_·6H_2_O. Until now, natural and synthetic carnallite, KCl·MgCl_2_·6H_2_O, was only known in its ortho­rhom­bic form [*a* = 16.0780 (3), *b* = 22.3850 (5), *c* = 9.5422 (2) Å and space group *Pnna*].

## Abstract 2

The previously unknown monoclinic form of potassium carnallite, KCl·MgCl_2_·6H_2_O, was found as a metastable phase occurring during crystallization studies of potash salt solutions.

## Introduction   

The natural evaporitic mineral carnallite, KCl·MgCl_2_·6H_2_O, is a main source for potash fertilizer production and for the production of magnesium chloride. Double salts with the general formula *MX*·*M*′*X*_2_·6H_2_O, where *M* is a large monovalent cation, *M*′ is a small divalent cation and *X* is Cl^−^, Br^−^ or I^−^, represent a family of structurally similar com­pounds. With the exception of *M* = K^+^ or Li(H_2_O)^+^, carnallite-type com­pounds crystallize with a monoclinic lattice (see Table 1 ▸). As can be seen in Table 1 ▸, the monoclinic angle for all com­pounds is near 90° and the respective basis plane is nearly square. Therefore, in older references, the cell was described as ‘tetra­gonal’ and ‘weak monoclinic’ (Andress & Saffe, 1939 ▸).

The structures of carnallites are typically described as perov­skite-like, with the halide anions arranged octa­hedrally around the monovalent cations. The corners of these octa­hedra are linked to neighbouring octa­hedra, forming a cubic lattice with large holes having cubocta­hedron geometry. The cations inside the cubocta­hedron have contact with 12 nearest equidistant anions. In the case of the carnallites, these are the divalent hy­drated *M*′ cations. Thus, the six water mol­ecules of *M*′ can form 12 hydrogen bonds with the halide anions. Small distortions lower the symmetry of the lattice (in the case of monoclinic carnallites), while the general arrangement of atoms remains.

An alternative description of the perovskite lattice is a cubic close-packed stacking of anions with cations (here *M*) occupying the octa­hedral holes. This is valid for all known carnallite-type structures, except for the mineral carnallite itself, where every third layer consists of a hexa­gonal stack denoted as *hcc*. The unit cell is enlarged to six layers, symbolized as a 6H structure. A hexa­gonal stack means the adjacent octa­hedra share faces. In this way, the mineral carnallite represents an exception to all other double salts of this stoichiometry.

Emons *et al.* (1988 ▸) applied the concept of a ‘tolerance factor, *t*′, known from oxidic perovskites *AB*O_3_ (Wells, 1984 ▸) to the carnallite-type. With
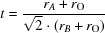
and setting *r_A_* = *r_M_*_’_, *r_B_* = *r_M_* and *r*_O_ = *r*_*X*_, the authors showed that with reasonable values of the radii, the factor *t* should not exceed values of about 1.045 to 1.061. The values obtained for KBr·MgBr_2_·6H_2_O and KCl·MgCl_2_·6H_2_O are 1.045 and 1.061, respectively. The bromide salt is monoclinic and the chloride exhibits a hexa­gonal stack with a threefold larger ortho­rhom­bic unit cell. Thus, the combination of potassium and magnesium with chloride is at the border of stability of pure cubic close packing. In the case of the smaller sodium ion, a much larger value is obtained, which explains why this type of double salt with sodium does not exist.

The K–Mg–Cl carnallite can form mixed crystals with corresponding (NH_4_)–Mg–Cl and K–Mg–Br double salts. Already at relatively small amounts of ammonium or bromide, the structure changes from ortho­rhom­bic to monoclinic.

Thermoanalytical investigations revealed solid-solid phase transitions for (NH_4_)Br·MgBr_2_·6H_2_O, RbBr·MgBr_2_·6H_2_O (Emons *et al.*, 1991 ▸) and CsCl·MgCl_2_·6H_2_O (Emons *et al.*, 1987 ▸). An endothermic effect at 435 K for RbBr·MgBr_2_·6H_2_O was suggested to be caused by an impurity of MgBr_2_·6H_2_O (Emons *et al.*, 1991 ▸). Dinnebier *et al.* (2008 ▸) recorded tem­per­ature-dependent powder X-ray diffraction patterns, show­ing that RbBr·MgBr_2_·6H_2_O undergoes a reversible transi­tion to a cubic perovskite structure, where the [Mg(H_2_O)_6_] octa­hedron is fourfold disordered. The temperature dependence of the lattice parameters of the analogous caesium com­pound showed a kink at around 430 K, but no symmetry break, which is in agreement with the absence of a thermal effect. Later, the same authors described the powder pattern of the high-temperature form of RbBr·MgBr_2_·6H_2_O by means of a rigid-body rotation of the [Mg(H_2_O)_6_] octa­hedra.

A survey on hydrated double salts *MX*·*M*′*X*_2_·*n*H_2_O (*M* = K^+^, NH_4_^+^, Rb^+^ or Cs^+^; *M*′ = Mg^2+^, Mn^2+^, Fe^2+^, Co^2+^, Ni^2+^ or Cu^2+^; *X* = Cl^−^, Br^−^ or I^−^) was given by Balarew & Tepavitcharova (2003 ▸). The authors discussed the solid phase structure and coordination in relation to the com­plexes and the concentration expected in saturated solutions.

The reason for the present investigation was the observation of a particular crystallization behaviour in brine samples from an underground nuclear repository in Morsleben (Niedersachsen, Germany). The brines were nearly saturated with carnallite, but during evaporation in laboratory dishes at room temperature, instead of the expected typical pseudo-hexa­gons of carnallite mineral, crystals of prismatic, nearly cubic, morphology were observed. In the same mine, Siemann (1994 ▸) and Herbert *et al.* (1995 ▸) discovered ammonium-bearing carnallite with a monoclinic lattice. However, preliminary investigations regarding brine com­position by ion chromatography excluded the presence of ammonium in the brine; furthermore, no bromide was found in the crystal. Selection of a single crystal and performance of a preliminary lattice determination suggested the presence of a new phase.

## Experimental   

### Synthesis and crystallization   

**Figure 1:**
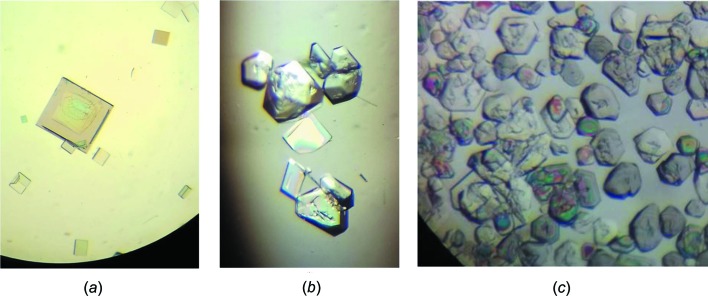
Microscope photos of monoclinic crystals (left), along with the pseudo-hexa­gonal carnallite crystallites formed over time (middle). Eventually, monoclinic KCl·MgCl_2_·6H_2_O dissolves com­pletely and the well-known pseudo-hexa­gonal forms remain (right). Crystallite edge lengths are on average between 0.1 and 0.5 mm.

To reproduce the crystallization of the unknown phase from natural brine, a series of pure synthetic solutions (4.1 molal MgCl_2_ + 0.2 molal KCl/4.7 molal MgCl_2_ + 0.05 molal KCl) were prepared and evaporated slowly over a period of 2–4 d in quiescent crystallization dishes (about 5 ml) at room temperature. Since it was speculated that impurities could influence nucleation, the addition of bromide (0.0025 to 0.01 molal) and sulfate (0.01 to 0.04 molal) was used to simulate the impurities of the natural Morsleben mine samples. Two samples were not spiked with bromide or sulfate. While sulfate will not be incorporated into the solid phase, bromide is expected to form a solid solution with the chloride in the carnallite. However, the distribution coefficient of bromide between carnallite and its solution in mass% has a value of about 0.5 (Braitsch, 1962 ▸), which with an impurity level of 0.01 mol kg^−1^ H_2_O (0.6 mass% Br^−^) yields a maximum content of 0.3 mass% Br^−^ in the carnallite. The latter corresponds to about 0.15 mol% Br^−^ in the carnallite. Nine evaporation experiments (starting solution com­positions are listed in Table 2 ▸) were performed, confirming the observation that the plate-like nearly square (monoclinic) morphology appears first or simultaneously with the pseudo-hexa­gonal carnallite. This was true for all solutions, both the spiked and unspiked. After some hours, the monoclinic form dissolves and the known pseudo-hexa­gonal form remains (see photographs in Fig. 1 ▸). Podder *et al.* (2013 ▸) also observed ‘quadratic plates’ in their experiments to grow carnallite crystals; however, they hypothesized the presence of a type of surface-grown ‘Hopper crystals’. Contrary to Podder *et al.*, we also observed the growth of square crystals on the bottom of the dishes. Thus, surface-influenced nucleation cannot be considered as crucial to obtain the monoclinic form of carnallite.

### Single-crystal analysis and refinement   

Crystal data, data collection and structure refinement details are summarized in Table 3 ▸. For single-crystal diffraction with a Stoe IPDS II image-plate diffraction system, a suitable crystal was selected under the microscope in polarized light. The crystal was fixed by high-purity silicon grease on a 0.1 mm diameter Hilgenberg glass capillary and after determination of the monoclinic unit cell, a triclinic strategy based on this cell was applied for the measurement of the diffracted intensities at a temperature of 200 K.

A structure solution using direct methods and a refinement of the atomic positions led to the atomic coordinates of the K, Mg, O and Cl ions. The positions of the H atoms could be located from residual electron-density maxima after further refinement. Anisotropic displacement parameters were determined for the heavy atoms and refinement of isotropic displacement parameters was performed for H atoms.

Further analysis showed that there was a continuous drop in intensity during the measurement period, indicating the beginning of crystal decom­position. The drop in intensity was taken into account by applying a decay correction. Thus, the *R*_int_ value was reduced from 8.00 to 4.58%.

An attempt to perform chemical analysis on single crystallites using SEM–EDX (scanning electron microscopy with energy dispersive X-ray analysis) did not lead to a representative result, as the adherent mother liquor could not be com­pletely removed. Hence, slightly higher magnesium and chloride contents were always measured in com­parison to potassium. However, structure analysis revealed an unambiguous K:Mg:Cl ratio of 1:1:3 in the crystal, which crystallized from a solution containing only KCl and MgCl_2_ (No. 2 in Table 2 ▸).

## Results and discussion   

**Figure 2:**
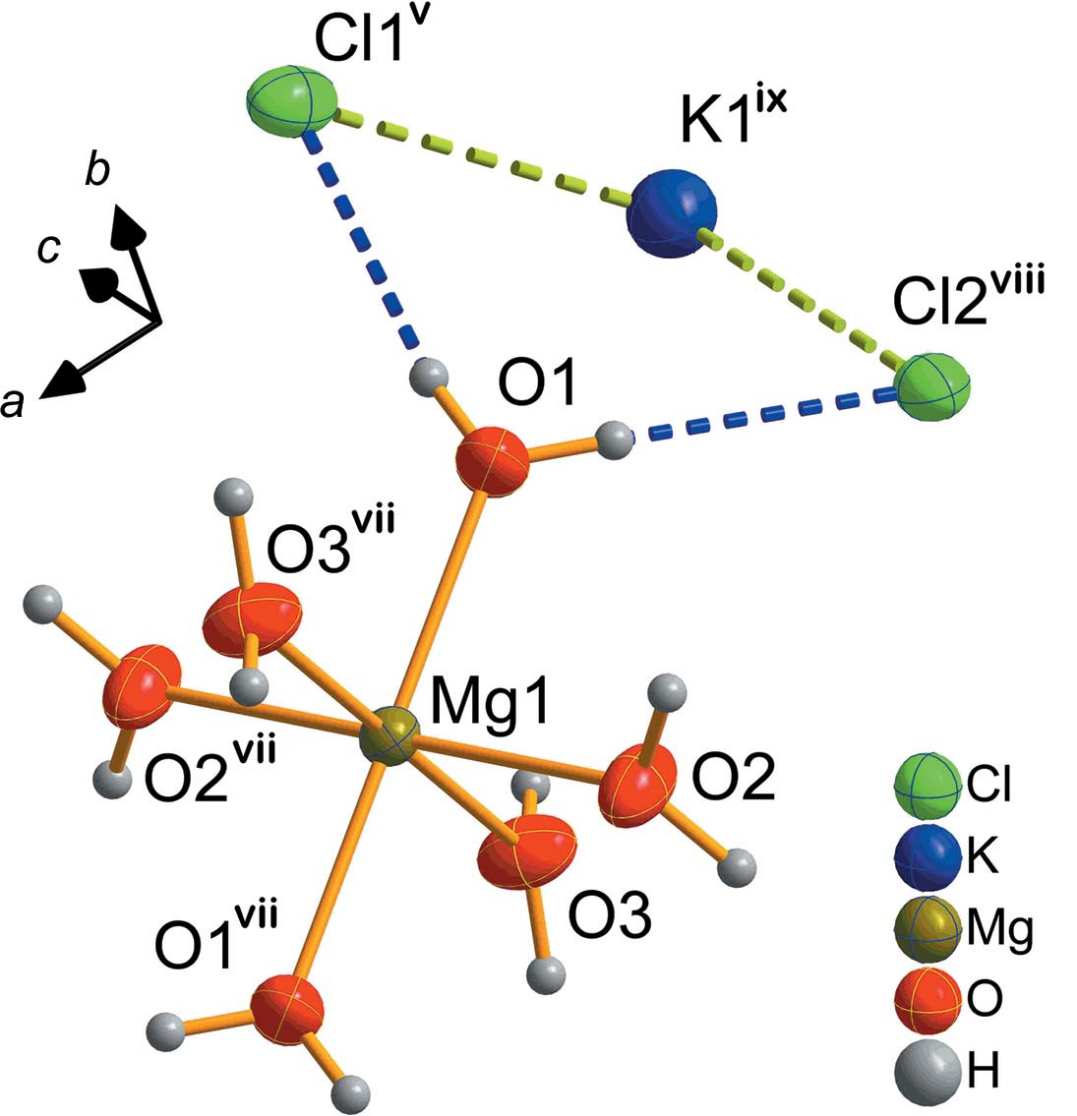
The asymmetric unit and symmetry-related atoms of KCl·MgCl_2_·6H_2_O. Displacement ellipsoids are drawn at the 50% probability level and H atoms are not labelled. [Symmetry codes: (v) *x* + 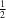, −*y* + 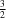, *z* + 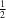; (vii) −*x* + 2, −*y* + 1, −*z* + 1; (viii) −*x* + 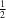, *y* − 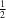, −*z* + 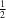; (ix) −*x* + 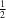, −*y* + 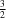, −*z* + 1.]

Contrary to ortho­rhom­bic carnallite, the structure of the new monoclinic form of KCl·MgCl_2_·6H_2_O contains only one crystallographic position for Mg and one for K, as shown in Fig. 2 ▸. The structure, which is isotypic with all other monoclinic carnallite-type double salts, displays a distorted perovskite-like lattice with corner-linked KCl_6_ octa­hedra and single Mg(H_2_O)_6_ octa­hedra in its large 12-fold coordinated holes.

**Figure 3:**
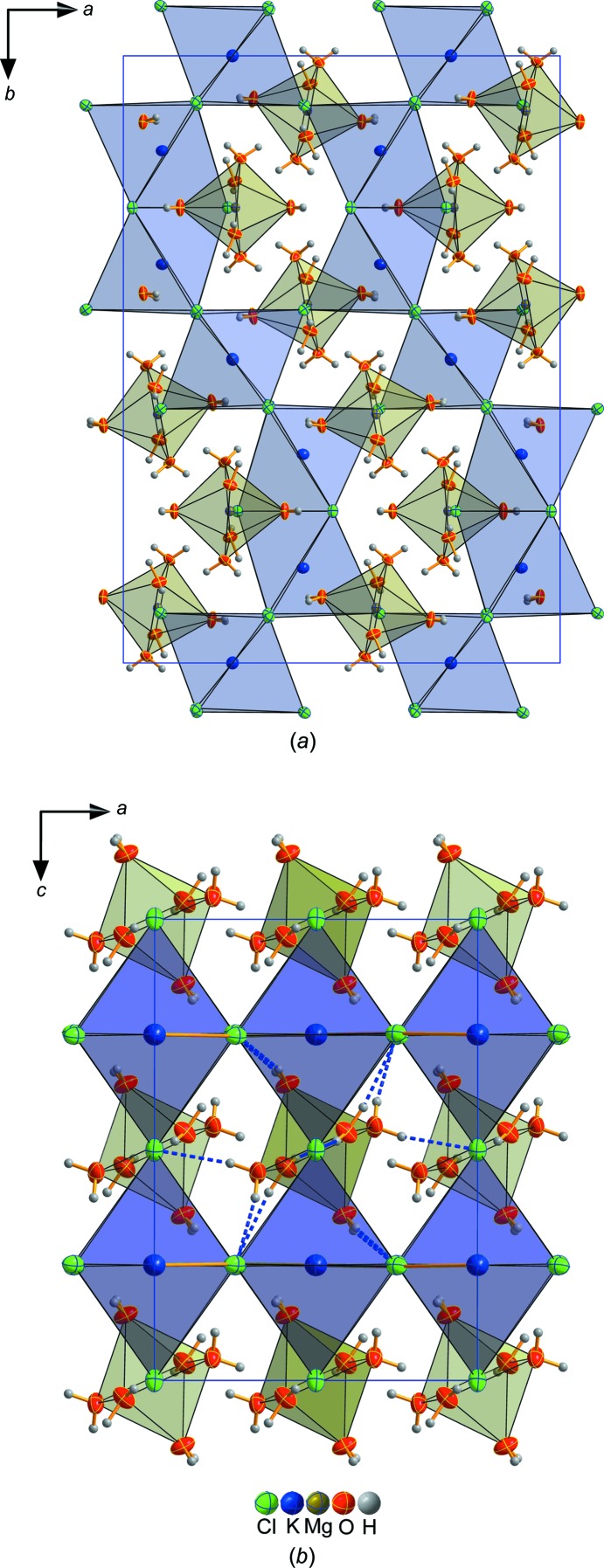
Comparison of the unit cells of (*a*) ortho­rhom­bic and (*b*) monoclinic KCl·MgCl_2_·6H_2_O.

**Figure 4:**
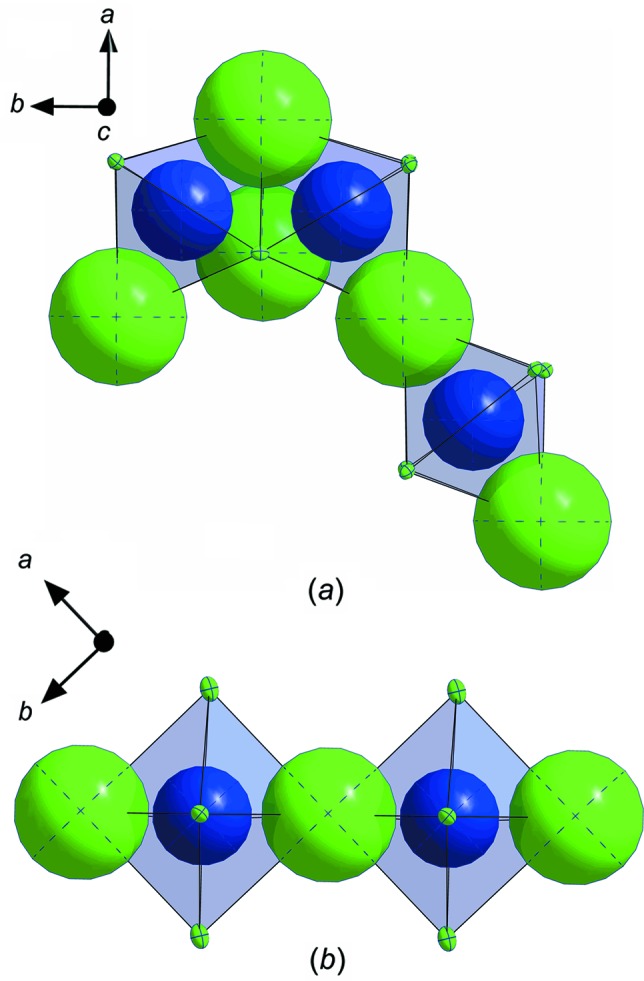
Comparison of the K–Cl octa­hedra and their connectivity in (*a*) ortho­rhom­bic and (*b*) monoclinic KCl·MgCl_2_·6H_2_O. Colour key: K blue and Cl green.

In Fig. 3 ▸, the unit cells of ortho­rhom­bic and monoclinic carnallite are com­pared. In the ortho­rhom­bic structure, two thirds of the KCl_6_ octa­hedra are face-linked, resulting in a 6H unit cell. Fig. 4 ▸ emphasizes this coordination for potassium and chloride ions.

While packing considerations between cations and anions are important for structure formation, the strengths of hydrogen bonds (bond lengths and angles) should also have a significant effect. Table 4 ▸ lists the hydrogen bonds with bond lengths between 2.28 (4) and 2.44 (3) Å. However, the energetic balance between the various types of inter­actions are delicate. There is no easy argument for a decision regarding the predominant effect. For example, one should expect larger bond distances in the metastable monoclinic form in com­parison to the stable ortho­rhom­bic form; however, adding all H⋯Cl hydrogen-bond lengths from the water mol­ecules of the two crystallographically distant Mg(H_2_O)_6_ octa­hedra in the ortho­rhom­bic structure yields a value of 57.44 Å, whereas double the same distance for Mg1 in the monoclinic structure gives 56.28 Å. The mean Mg—O distances in the ortho­rhom­bic and monoclinic structures are 2.044 and 2.047 Å, respectively.

The observation that the monoclinic form dissolves after some time proves its metastability. Metastable phases should possess lower densities than the stable forms. Our calculated density at 200 K is 1.586 Mg m^−3^, while Schlemper *et al.* (1985 ▸) calculated a value of 1.60 Mg m^−3^ at room temperature for the ortho­rhom­bic form. The experimental density determination of Andress & Saffe (1939 ▸) gave a value of 1.602 Mg m^−3^. Thus, our density value (even if only slightly lower) confirms the metastability of monoclinic KCl·MgCl_2_·6H_2_O. The small density difference cannot be easily recognized when com­paring some selected bond lengths.

With regard to the faster crystallization of the metastable phase (Ostwald’s step rule), the variability of the Mg(H_2_O)_6_ octa­hedra in the two carnallite structures also provides a possible explanation. All O—Mg—O angles in the metastable monoclinic structure are very close to 90° [between 89.90 (7) and 90.10 (7)°], while in the ortho­rhom­bic structure, according to the data of Schemper *et al.*, the Mg(H_2_O)_6_ octa­hedra are more distorted (89.46–94.83° for Mg1 and 88.71–91.48° for Mg2). Undistorted Mg(H_2_O)_6_ octa­hedra, as they exist in solution, are incorporated more quickly into a crystal lattice with only minimal changes. For the formation of more distorted geometries, even with an energetically balanced arrangement over the whole lattice, longer crystallization times are required.

## Conclusion   

Crystallization experiments with natural and synthetic potash salt brines revealed the formation of a new phase of potassium carnallite, *i.e.* KCl·MgCl_2_·6H_2_O. Crystal structure analysis yielded a monoclinic form of potassium carnallite, for which the crystal structure was solved. The monoclinic form represents the stable form of all other members of the carnallite family (except lithium carnallite), but was missing for potassium carnallite until now. The radii of K^+^, [Mg(H_2_O)_6_]^2+^ and Cl^−^ in KCl·MgCl_2_·6H_2_O are in the border region of stability for the simple cubic close-packed anion stacking similar to a perovskite lattice. However, nucleation and growth of the ‘normal’ monoclinic potassium carnallite can be observed in brines. The phase is metastable and transforms *via* a dissolution–crystallization mechanism into the stable ortho­rhom­bic form after some time. Since the nucleation and crystallization kinetics represent an important issue in industrial crystallization, future investigation of the kinetics should consider the possible formation of monoclinic carnallite.

## Supplementary Material

Crystal structure: contains datablock(s) I, global. DOI: 10.1107/S2053229620005197/uk3195sup1.cif

Structure factors: contains datablock(s) I. DOI: 10.1107/S2053229620005197/uk3195Isup2.hkl

CCDC reference: 1984700

## Figures and Tables

**Table 1:** Survey of the crystallographic data of carnallite-type double salts

Formula	Space group	Cell axes (Å)			β	*Z*	X-ray^*a*^	Reference
[Li(H_2_O)][Mg(H_2_O)_6_]Cl_3_	*C*2/*m*	9.2	9.7	13.4	93.3		P	Emons *et al.* (1988 ▸)
	*R*3	9.2		12.0		3	SC	Schmidt *et al.* (2009 ▸)
K[Mg(H_2_O)_6_]Cl_3_	*Pbnn*	9.6	16.0	22.6				Leonhardt (1930 ▸)
	*Pban*	9.5	16.0	22.5		12	SC	Andress & Saffe (1939 ▸)
	*Pnna*	9.6	16.1	22.6			SC	Fischer (1973 ▸)
	*Pnna*	16.1	22.5	9.5		12	SC	Schlemper *et al.* (1985 ▸)
Rb[Mg(H_2_O)_6_]Cl_3_	*P*4/*n*	13.3	6.6	6.6		2	SC	Andress & Saffe (1939 ▸)
	*C*2/*c*	9.3	9.5	13.3	90.2	4	SC^*b*^	Marsh (1992*a* ▸)
	*C*2/*c*	9.3	9.6	13.3	90.4		P	Emons *et al.* (1988 ▸)
(NH_4_)[Mg(H_2_O)_6_]Cl_3_	*P*4/*n*	13.3	6.7	6.7		2	SC	Andress & Saffe (1939 ▸)
	*C*2/*c*	9.3	9.6	13.3	90.1	4	SC	Solans *et al.* (1983 ▸)
	*C*2/*c*	9.3	9.5	13.3	90.1	4	SC^*b*^	Marsh (1992*b* ▸)
Cs[Mg(H_2_O)_6_]Cl_3_	*C*2/*c*	9.4	9.6	13.3	90.3		P	Emons *et al.* (1988 ▸)
K[Mg(H_2_O)_6_]Br_3_	*P*4/*n*	13.6	6.8	6.8			SC	Andress & Saffe (1939 ▸)
Rb[Mg(H_2_O)_6_]Br_3_	*C*2/*c*	9.6	9.8	13.8	90.1		P	Emons *et al.* (1988 ▸)
	*C*2/*c*	9.6	9.9	13.8	90.1	4	P	Dinnebier *et al.* (2008 ▸)
*T* ≥ 85 °C	*Pm* 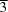 *m*	6.94	6.94	6.94		1	P	Dinnebier *et al.* (2008 ▸)
(NH_4_)[Mg(H_2_O)_6_]Br_3_	*C*2/*c*	9.6	9.8	13.7	90.2		P	Emons *et al.* (1988 ▸)
Cs[Mg(H_2_O)_6_]Br_3_	*C*2/*c*	9.8	9.9	13.9	90.1		P	Emons *et al.* (1988 ▸)
	*C*2/*c*	9.8	10.0	14.0	90.1	4	P	Dinnebier *et al.* (2008 ▸)
K[Mg(H_2_O)_6_]I_3_	*C*2/*c*	10.0	10.2	14.4	90.1		P	Emons *et al.* (1988 ▸)
Rb[Mg(H_2_O)_6_]I_3_	*C*2/*c*	10.0	10.3	14.5	90.6		P	Emons *et al.* (1988 ▸)
(NH_4_)[Mg(H_2_O)_6_]I_3_	*C*2/*c*	10.0	10.2	14.3	90.4		P	Emons *et al.* (1988 ▸)
Cs[Mg(H_2_O)_6_]I_3_	Compound not known							
(NH_4_)[Fe(H_2_O)_6_]Br_3_	Synthesis only							Mercier (1937 ▸)
K[Ni(H_2_O)_6_]Br_3_	*C*2/*c*	9.5	9.7	13.6	90.1	4	SC	Tepavitcharova *et al.* (1997 ▸)
(NH_4_)[Ni(H_2_O)_6_]Br_3_	*C*2/*c*	9.6	9.8	13.7	90.2	4	SC	Tepavitcharova *et al.* (1997 ▸)
Rb[Co(H_2_O)_6_]Br_3_	*C*2/*c*	9.6	9.8	13.7	90.1	4	SC	Tepavitcharova *et al.* (1997 ▸)
(NMe_4_)[Mg(H_2_O)_6_]Br_3_	*P*2_1_2_1_2_1_	7.7	9.4	22.5			SC	Gusev *et al.* (2011 ▸)
								
Mixed crystals								
(NH_4_)_*x*_K_(1−*x*)_[Mg(H_2_O)_6_]Cl_3_								
*x* = 0.33							P	Siemann (1994 ▸)
*x* = 0.36	*P*2/*c* or *Pc*	6.7	6.7	13.2	90			Herbert *et al.* (1995 ▸)
*x* = 0.5	*C*2/*c*	9.3	9.5	13.2	90.2	4	SC	Okrugin *et al.* (2019 ▸)
*x* = 0.52		9.3	9.6	13.3	90.1		SC	Yang *et al.* (2019 ▸)
K[Mg(H_2_O)_6_][Br_*x*_Cl_(1−*x*)_]_3_								
0 < *x* < 0.12	Rhombic–pseudohexa­gonal						SC	Andress & Saffe (1939 ▸)
0.12 < *x* < 0.85	Tetra­gonal						SC	Andress & Saffe (1939 ▸)
0.85 < *x* < 1.0	Rhombic–pseudo­tetra­gonal						SC	Andress & Saffe (1939 ▸)

Notes: (*a*) P = powder XRD and SC = single-crystal XRD; (*b*) recalculated from the triclinic cells given by Waizumi *et al.* (1991 ▸).

**Table 2:** Initial solution com­positions (*m* in mol kg^−1^ H_2_O) of the nine eva­por­ation experiments

No.	*m*(Mg^2+^)	*m*(K^+^)	*m*(Cl^−^)	*m*(SO_4_^2−^)	*m*(Br^−^)
1	4.086	0.192	8.300	0	0
2	4.630	0.050	9.326	0	0
3	4.70	0.054	9.45	0	0.0028
4	4.70	0.057	9.45	0	0.0055
5	4.70	0.059	9.45	0	0.0074
6	4.70	0.062	9.45	0	0.0106
7	4.725	0.069	9.509	0.011	0
8	4.753	0.089	9.451	0,.21	0
9	4.735	0.108	9.32	0.030	0

**Table 3:** Experimental details

Crystal data	
Chemical formula	KCl·MgCl_2_·6H_2_O
*M*_r_	277.86
Crystal system, space group	Monoclinic, *C*2/*c*
Temperature (K)	200
*a*, *b*, *c* (Å)	9.251 (2), 9.516 (2), 13.217 (4)
β (°)	90.06 (2)
*V* (Å^3^)	1163.6 (5)
*Z*	4
Radiation type	Mo *K*α
μ (mm^−1^)	1.19
Crystal size (mm)	0.3 × 0.26 × 0.06

Data collection	
Diffractometer	Stoe IPDS 2T
Absorption correction	Integration Coppens (1970 ▸)
*T*_min_, *T*_max_	0.665, 0.983
No. of measured, independent and observed [*I* > 2σ(*I*)] reflections	6028, 1341, 1096
*R*_int_	0.046
(sin θ/λ)_max_ (Å^−1^)	0.650

Refinement	
*R*[*F* ^2^ > 2σ(*F* ^2^)], *wR*(*F* ^2^), *S*	0.035, 0.096, 1.21
No. of reflections	1341
No. of parameters	78
H-atom treatment	All H-atom parameters refined
Δρ_max_, Δρ_min_ (e Å^−3^)	0.25, −0.49

Computer programs: *X-AREA* (Stoe & Cie, 2002 ▸), *X-RED32* (Stoe & Cie, 2002 ▸), *SHELXT* (Sheldrick, 2015*a* ▸), *SHELXL2016* (Sheldrick, 2015*b* ▸) and *DIAMOND* (Brandenburg, 2017 ▸).

**Table 4:** Hydrogen-bond geometry (Å, °)

*D*—H⋯*A*	*D*—H	H⋯*A*	*D*⋯*A*	*D*—H⋯*A*
O3—H3*B*⋯Cl2^i^	0.81 (3)	2.34 (3)	3.1345 (18)	172 (3)
O3—H3*A*⋯Cl1^ii^	0.82 (3)	2.36 (3)	3.1703 (19)	167 (3)
O2—H2*B*⋯Cl1^iii^	0.89 (4)	2.31 (4)	3.1689 (18)	164 (3)
O2—H2*A*⋯Cl1^iv^	0.86 (4)	2.28 (4)	3.1369 (18)	172 (3)
O1—H1*B*⋯Cl1^v^	0.81 (4)	2.35 (4)	3.1500 (18)	171 (4)
O1—H1*A*⋯Cl2^vi^	0.75 (3)	2.44 (3)	3.1863 (19)	171 (3)

Symmetry codes: (i) 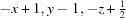; (ii) 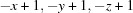; (iii) 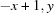, 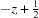; (iv) 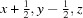; (v) 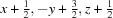; (vi) 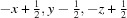.
